# A photoactivation-free viability PCR reagent for rapid, culture-independent detection of live bacteria

**DOI:** 10.1128/spectrum.03935-25

**Published:** 2026-02-27

**Authors:** Nathan Feirer, Matthew Poole, Nishanth Kuchi, Caitlin Hawthorn, Rebecca Marino, James J. Cali, Attiq Rehman, Diane Botelho, Subhanjan Mondal

**Affiliations:** 1 Promega Corporation, Fitchburg, Wisconsin, USA; 2 Research and Productivity Council (RPC), Moncton, New Brunswick, Canada; 3 PACE Analytical, Pittsburgh, Pennsylvania, USA; 4 Research and Productivity Council (RPC), Fredericton, New Brunswick, Canada; Connecticut Agricultural Experiment Station, New Haven, Connecticut, USA

**Keywords:** *Legionella*, viability PCR, qPCR, bacterial viability

## Abstract

Detection of viable bacteria in complex samples is a long-standing challenge in microbiology and public health. Traditional culture methods are slow and often fail to capture viable but non-culturable cells, while PCR-based methods can overestimate bacterial loads by amplifying DNA from non-viable (dead) cells. To address this challenge, we evaluated a novel, photoactivation-free viability PCR (vPCR) reagent from Promega Corporation that selectively crosslinks DNA from non-viable cells, rendering it unamplifiable by qPCR. This approach enables accurate, culture-independent quantification of viable bacteria. We optimized key parameters in both gram-positive (*Listeria*) and gram-negative (*Pseudomonas* and *Legionella*) bacteria, including reagent concentration, amplicon length, and workflow format to ensure robust viability discrimination. We demonstrate that this method detects reductions in viable *Legionella* following heat treatment and is compatible with filter-concentrated samples. This is the first description of a chemically defined, non-photoactivated vPCR reagent and its successful application to rapid, accurate detection of viable *Legionella*, with implications for building water safety and public health surveillance.

**IMPORTANCE:**

Rapid and reliable detection of viable bacteria is essential for protecting public health, monitoring water quality, ensuring food safety, and advancing clinical diagnostics. Conventional culture methods underestimate viable cells and require days to deliver results, while conventional PCR-based tests cannot distinguish viable (live) from non-viable (dead) organisms. Here, we describe a photoactivation-free viability PCR reagent that overcomes these limitations by selectively blocking PCR amplification from non-viable cells. Using *Legionella pneumophila*, a waterborne pathogen that causes severe pneumonia, we show that this method provides accurate, culture-independent measurements of viable bacteria in water samples within hours. Results align with culture-based methods while avoiding long delays and technical drawbacks of traditional approaches. By simplifying workflows and improving accuracy, this reagent offers a broadly applicable tool for detecting viable microbes, enabling faster interventions and supporting microbial testing across environmental, clinical, and industrial settings.

## INTRODUCTION

Accurate detection of viable bacteria remains a critical challenge in microbiology and public health. Traditional culture-based methods rely on the ability of bacteria to grow on agar plates, which excludes a significant portion of the microbial community (1), particularly those in a viable but non-culturable (VBNC) state (2–4). Consequently, culture-based enumeration often underestimates viable cells and delays actionable results, especially in environmental and clinical settings (5, 6). Although alternative techniques such as microscopy (7, 8), ATP measurements (9), RNA analysis (10), and flow cytometry (11) offer culture-independent insights into bacterial viability, they are generally low throughput, technically demanding, cost prohibitive, or poorly suited to field applications.

**Fig 1:**
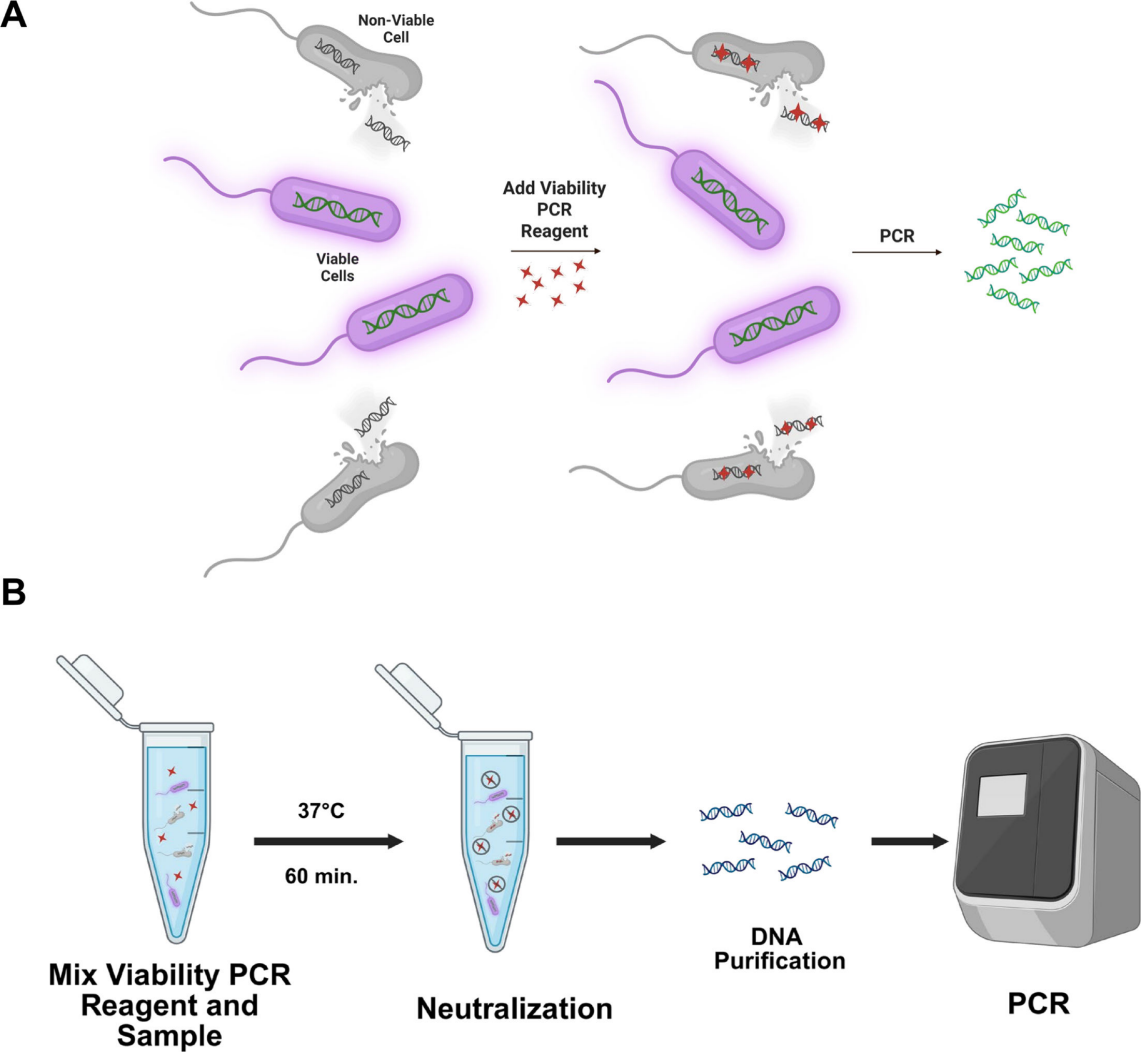
Viability PCR concept and workflow. (**A**) Viability PCR Reagent (red diamonds) penetrates only dead cells (gray), binding and modifying their DNA to prevent amplification, ensuring the PCR signal originates predominantly from live cells (purple) with intact membranes. (**B**) Photoactivation-independent viability PCR workflow. The sample is treated with Viability PCR Reagent, incubated at 37°C, followed by chemical neutralization of unreacted reagent. DNA is purified, and PCR is performed using bacterial-specific primers. Images created using www.biorender.com.

For the last three decades, conventional PCR (without any sample pretreatment) has been a widely used molecular technique for detecting human pathogens (12–15). However, a major limitation of this approach is the amplification of DNA originating from non-viable organisms, which often leads to overestimation of viable pathogen load (16). To overcome this limitation, viability PCR (vPCR) has emerged as a promising tool for differentiating viable from non-viable bacteria (16, 17). In vPCR, membrane-impermeable nucleic acid-binding reagents are used to selectively block amplification of DNA from non-viable or lysed cells, as well as from cell-free DNA (Fig. 1A), providing a more accurate estimate of viable organisms. This approach reduces the false-positive issue associated with conventional PCR by selectively amplifying DNA from cells with intact cell membranes (16, 17). Additionally, vPCR is particularly useful for monitoring the efficacy of common disinfection techniques such as heat treatment, chlorinated biocides, and benzalkonium, as all these methods affect the integrity of bacterial cell membranes (18–20). The core chemical component necessary for vPCR is a DNA modifying agent that is impermeable to intact, viable cell membranes. This reagent binds to free DNA or DNA from membrane-compromised cells and creates irreversible intra- or inter-strand covalent linkages within the DNA molecule. As a result of this permanent modification, the DNA is not amenable to subsequent amplification by PCR.

Ethidium monoazide (EMA) (21) and propidium monoazide (PMA) are well-established vPCR reagents (22). Both contain a phenanthridine ring moiety that intercalates into DNA and an azide group that upon blue light photoactivation, forms a nitrene radical that covalently modifies DNA (23). EMA and PMA reduce PCR signal from non-viable and/or membrane-compromised bacterial cells in various sample matrices (21, 24–29). While EMA/PMA-mediated vPCR has demonstrated utility, it has several limitations, including viable cell signal inhibition (30–32), cell concentration-dependent variability (33), and reduced performance in complex matrices (34–36).

In this report, we describe the evaluation of a novel vPCR workflow utilizing a nucleic acid-modifying reagent (Viability PCR Reagent [VR]) that does not require photoactivation. We demonstrate that this workflow effectively reduces qPCR signal from non-viable cells or cell-free DNA across a range of gram-positive and gram-negative bacteria. Using these bacterial systems, key optimization parameters such as reagent concentration and amplicon length are further explored. Finally, we demonstrate the applicability of this workflow in real-world, filter-concentrated *Legionella* samples collected from cooling towers (CTs).

## RESULTS

### Optimization of Viability PCR Reagent performance

**Fig 2:**
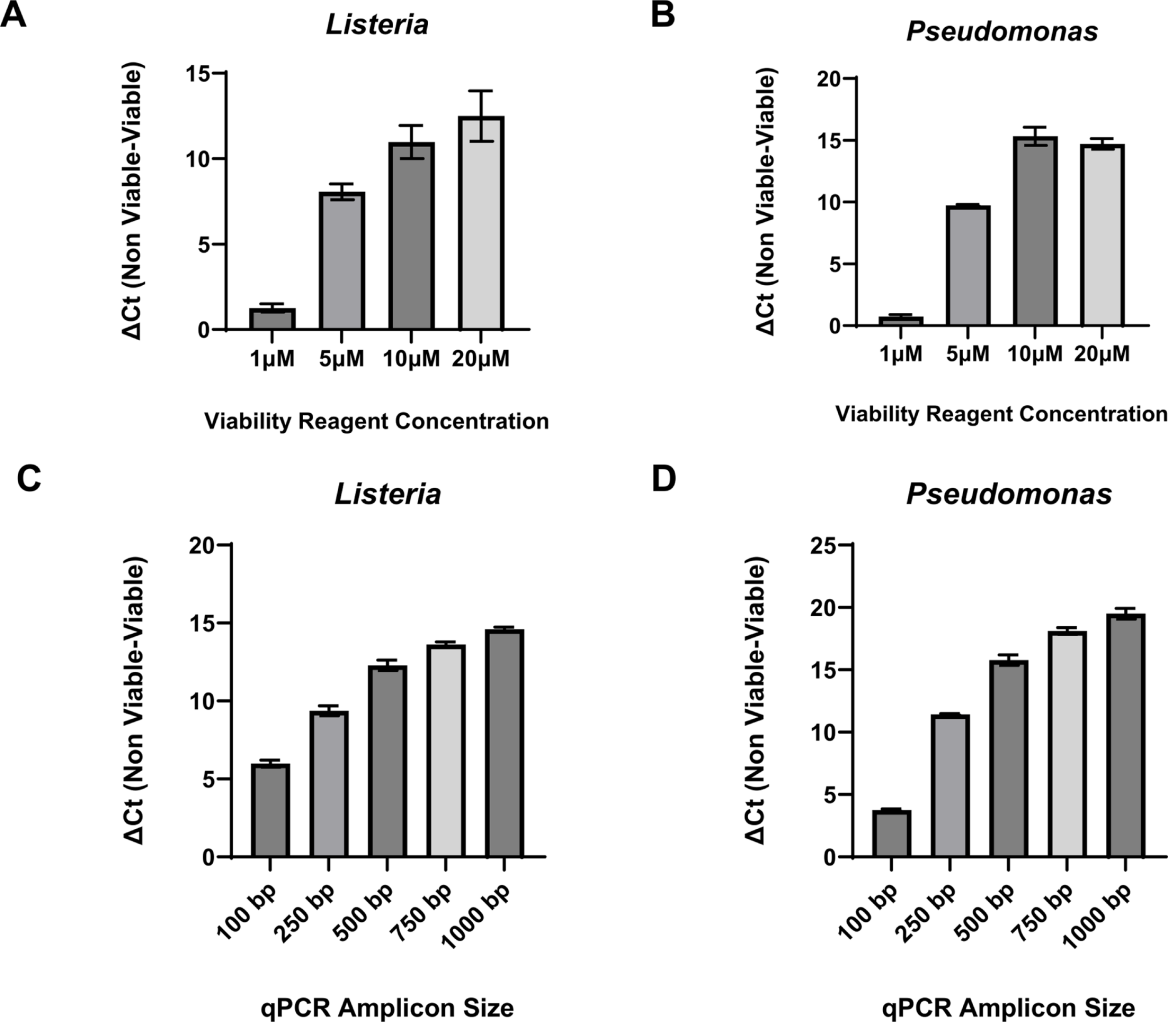
Optimization of vPCR workflow parameters. (**A and B**) Titrations of Viability PCR Reagent (VR). *Listeria innocua* (**A**) or *Pseudomonas aeruginosa* (**B**) (viable and heat killed) were treated with listed concentrations of VR, followed by neutralization and nucleic acid purification. Dye-based qPCR was performed using bacterial-specific primers to amplify ~500 bp templates. (**C and D**) Effect of qPCR amplicon length on viability PCR performance. *Listeria* (**C**) or *Pseudomonas* (**D**) (viable and heat killed) were treated with 10 µM of VR, followed by neutralization and nucleic acid purification. Dye-based qPCR was performed using bacterial-specific primers amplifying DNA fragments of the indicated sizes. The ΔC_t_ value is defined as the cycle threshold (C_t_) difference between live and heat-killed bacterial cells. Data represented as mean ± standard deviation from three independent replicates.

A key goal of our study was to develop a novel vPCR workflow incorporating a chemical agent that demonstrates both impermeabilities to viable cell membranes and potent nucleic acid modification activity that does not require photoactivation. To achieve this goal, the Viability PCR Reagent System (Promega Corporation, Madison, WI, USA) was evaluated for further optimization and evaluation due to its ability to suppress non-viable cell qPCR signal without requiring photoactivation. The general workflow of this technology is demonstrated in Fig. 1B. During our optimization efforts, heat-killed bacterial cells acted as a proxy for “non-viable” cells. Heat-killed cells are not viable for growth but do not exhibit completely ruptured membranes (37). First, we tested a range of VR concentrations against both viable and non-viable *Listeria innocua* and *Pseudomonas aeruginosa*. Each bacterial species was treated with final concentrations of VR ranging from 1 to 20 µM. For *L. innocua*, the 10 and 20 µM treatment concentrations resulted in the highest ΔC_t_ (C_t-Non-viable_ − C_t-Viable_) values (Fig. 2A), with no significant difference between the two concentrations (*P* value: 0.21, two-tailed unpaired *t*-test, *n* = 3). *P. aeruginosa* exhibited a similar pattern, with ΔC_t_ values plateauing at the 10 and 20 µM treatment concentrations (Fig. 2B).

Another key optimization parameter in any vPCR assay is qPCR amplicon size (38). Accordingly, we reasoned that an increase in amplicon length would result in a subsequent increase in ΔC_t_ value due to more available sites for VR binding and modification. To test the relationship between amplicon length and ΔC_t_ value, viable and non-viable *L. innocua* and *P. aeruginosa* were treated with a constant concentration (10 µM) of VR, and qPCR was performed with five different amplicon sizes (100, 250, 500, 750, and 1,000 bp). For *L. innocua*, each increase in amplicon size resulted in a significant increase in ΔC_t_ value (*P* values: <0.05, two-tailed unpaired *t*-test, *n* = 3) (Fig. 2C). However, the rate of ΔC_t_ value increase was much higher, from 100 to 500 bp compared to 750–1,000 bp. *P. aeruginosa* exhibited a slightly different pattern, with the greatest ΔC_t_ value jump from 100 to 250 bp, with only small but statistically significant (*P* values: <0.05, two-tailed unpaired *t*-test, *n* = 3) incremental increases from 250 to 1,000 bp (Fig. 2D). These data suggest that optimal amplicon sizes for VR, depending on bacterial species, are between 250 and 500 bp. For both *Listeria* and *Pseudomonas*, the absolute ΔC_t_ values with PMAxx were similar to those obtained with the Viability PCR Reagent (Fig. S1A and B).

### vPCR for *Legionella*

With baseline optimization of the VR method complete, we aimed to demonstrate its applicability using a relevant microbial pathogen in a real-world setting. *Legionella pneumophila* is a Gram-negative waterborne pathogen that causes the bacterial pneumonia known as Legionnaires’ disease (39). *Legionella* infections commonly originate in man-made aquatic environments such as cooling towers, spas, and potable water systems (39). The current gold standard for assessing *Legionella* contamination in environmental samples is culture-based enumeration of *Legionella* (e.g., according to ISO 11731, *Water quality—Enumeration of Legionella*). Culture offers the advantage of quantifying only viable cells, providing environmental isolates for epidemiological investigations (40) and establishing threshold action limits for interventions (41). However, culturing requires 7–10 days to obtain results, exhibits substantial inter- and intra-laboratory variability (42), difficulty to recover stressed cells that may later regrow (VBNC [43–45]), often fails to isolate viable *Legionella* residing in amoeboid hosts (46), and requires culture confirmation and isolate identification to determine pathogenic species. These limitations hinder rapid assessment of building-level interventions and can lead to underestimation of risk.

Conventional PCR has been used to expedite the detection of *Legionella* in water samples; however, this approach lacks information regarding bacterial viability (15, 47). *Legionella* qPCR detection systems demonstrate excellent negative predictive value (NPV) in environmental monitoring but often greatly overestimate *Legionella* titer compared to traditional culture methods, limiting the ability to perform actionable interventions (48). For these reasons, vPCR is an intriguing tool for detecting viable *Legionella* cells, with PMA recently employed to assess *Legionella* contamination in evaporative cooling systems (49) and car washes (50).

To test the feasibility of using VR in vPCR for *Legionella* detection, we further evaluated VR within a *Legionella* qPCR workflow. To accomplish this, we used the GoTaq *Legionella* spp./*pneumophila*/SG1 qPCR Kit (Promega Corporation, Madison, WI, USA), a multiplex *Legionella* qPCR assay designed to simultaneously detect and quantify *L.* spp., *L. pneumophila*, and *L. pneumophila* serogroup 1 (SG1) (Fig. S2). In this kit, a 16S rRNA amplicon serves as a marker for *L.* spp., a *mip1* gene amplicon for *L. pneumophila*, and *wzm* for *L. pneumophila* SG1 (Fig. S2A). A linearized plasmid containing a firefly luciferase gene fragment is included as an internal positive control (IPC) to monitor assay inhibition. We also utilized the qPCR quantification standard provided in the kit to quantify the number of *Legionella* genomic copies in tested samples. All assays demonstrated acceptable performance, with linearities (*R*^2^ > 0.97) and qPCR efficiencies between 90% and 110% (Fig. S2B). We then evaluated the multiplexed qPCR assay specificity by testing both inclusivity and exclusivity. Seventeen *L. pneumophila* strains (including three *L. pneumophila* SG1) and an additional 20 non-*pneumophila L.* spp*.* were tested. The *L. pneumophila* (*mip1*) PCR was positive only for the 17 *L. pneumophila* strains; the *L. pneumophila* SG1 (*wzm*) PCR was only positive for the 3 *L. pneumophila* SG1 isolates; and the *L.* spp. (16S rRNA) PCR was positive for all *Legionella* strains (Table S3). As expected, the additional 28 non-*Legionella* microbes tested did not show amplification for all three *Legionella* targets (Table S4).

### On-filter *Legionella* vPCR workflow

**Fig 3:**
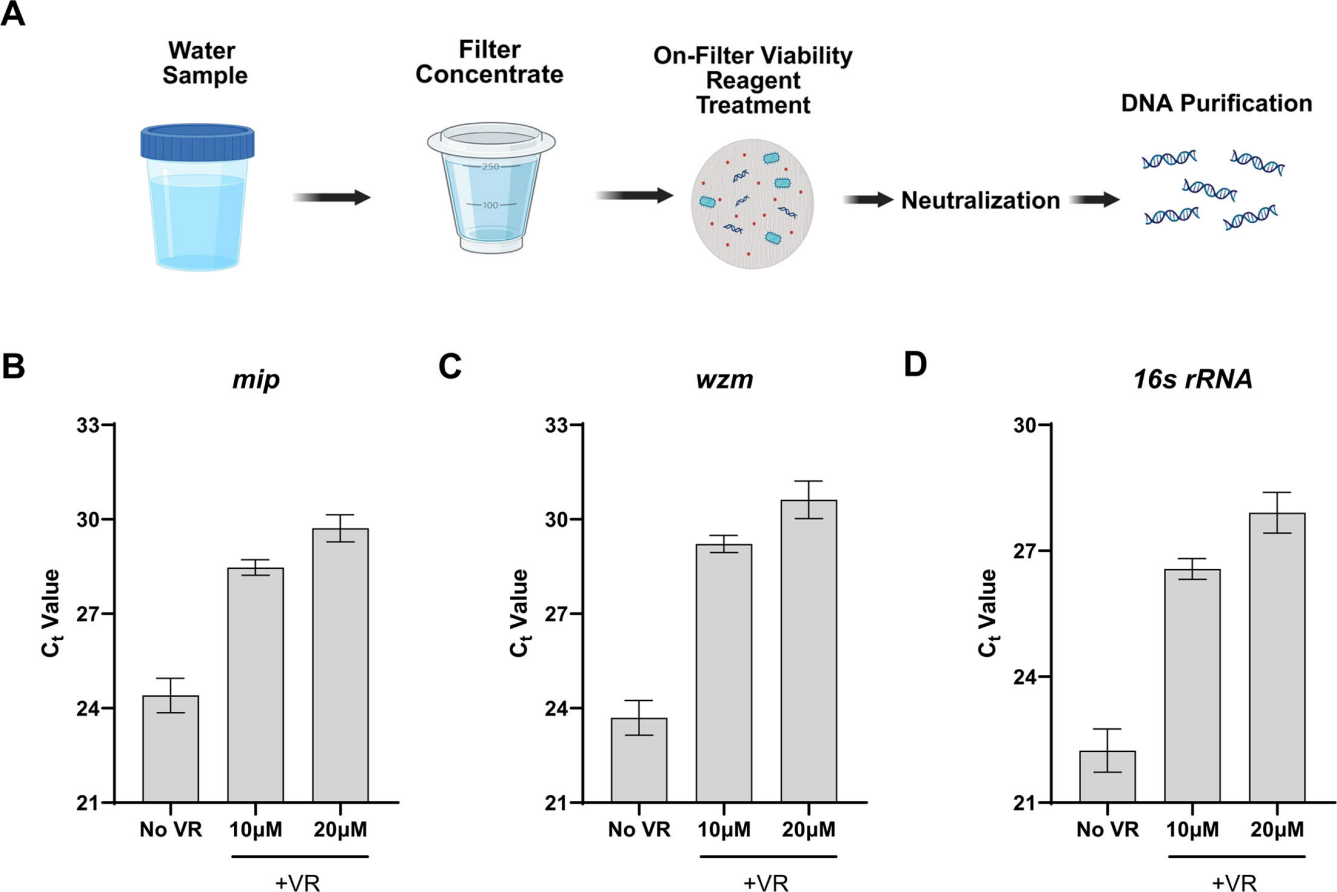
vPCR with filter-concentrated samples. (**A**) Workflow schematic for on-filter Viability PCR Reagent (VR) treatment. Bacterial cells are concentrated on polycarbonate filter disks, treated with Viability PCR Reagent. Following neutralization, automated nucleic purification is performed. (**B–D**) On-filter Viability PCR Reagent treatment of non-viable cells. Heat-treated (95°C, 15 min) *Legionella pneumophila* suspensions (10^5^ cells, 200 mL) were on-filter treated with 0 µM (no VR), 10 µM, or 20 µM as described in **A**. Following DNA purification, qPCR quantification of three separate *Legionella*-specific amplicons; (**B**) *mip*, (**C**) *wzm*, (**D**) *16s rRNA*, was performed using the GoTaq *Legionella* spp./*pneumophila*/SG1 described in Fig. S2. Error bars represent the standard deviation of triplicate filtrations for each condition. Images created using www.biorender.com.

*Legionella* cell concentrations in man-made aquatic environments are often low, typically in the range of 10^1^–10^3^ CFU/mL (51). Therefore, to increase detection sensitivity, larger volumes (100 mL–1 L) of water are commonly filter concentrated as part of routine sampling workflows (e.g., according to ISO 11731). To accommodate this sampling approach, we tested the functionality of the Viability PCR Reagent for treating *Legionella* directly on a filter (Fig. 3A). For this workflow, a mother culture of *L. pneumophila* (1 × 10^8^ CFU/mL) was heat treated at 95°C for 10 min and diluted to 1 × 10^3^ CFU/mL. The replicate 200 mL volumes of diluted sample were filtered through 0.2 µm polycarbonate filters, which were then incubated with either 0, 10, or 20 µM VR in 700 µL PBS for 1 h at 37°C. After incubation, VR was neutralized, and nucleic acids were extracted for analysis. Treatment of non-viable cells on the filter with VR significantly decreased *L. pneumophila* qPCR signal (*P* values: <0.05, two-tailed unpaired *t*-test, *n* = 3), with 10 µM VR (C_t_: 28.47) resulting in an over 1-log_10_ reduction of observed amplification signal (ΔC_t_: 4.06) compared to untreated filters (C_t_: 24.41) (Fig. 3B). Increasing the VR treatment concentration to 20 µM resulted in an additional twofold decrease in qPCR signal compared to the 10 µM treatment (ΔC_t_: 5.31). This trend of an additional twofold decrease for 20 µM vs 10 µM was consistent for the other two targets (*wzm* and 16sRNA) in the *Legionella* multiplex PCR assay (Fig. 3C and D).

### *Legionella* quantitation method comparison

**Fig 4:**
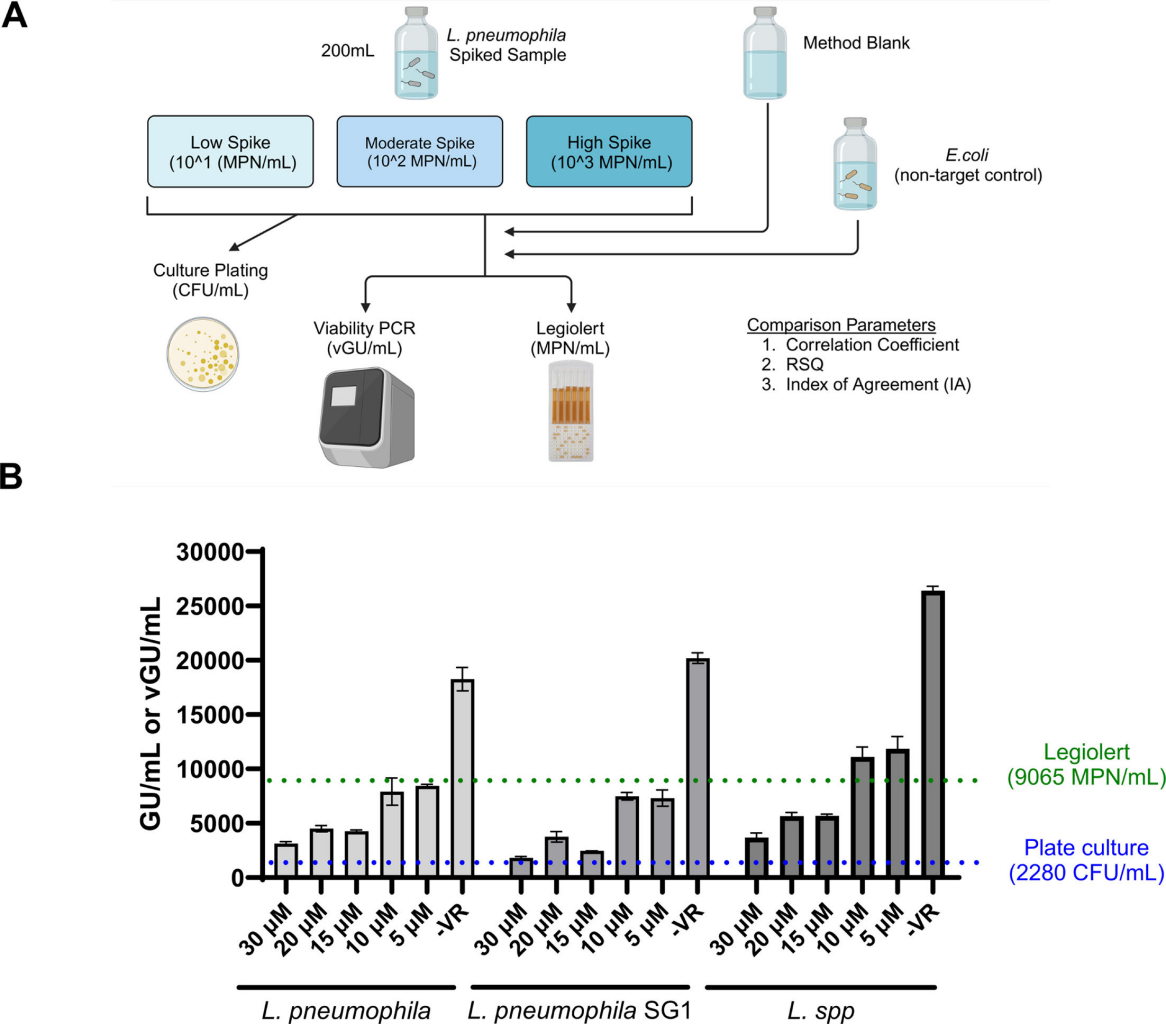
vPCR comparison to culture detection in spiked samples. (**A**) Experimental setup diagram of *Legionella* spike experiment. (**B**). vPCR results with “high spike” sample. Two hundred milliliters of *L. pneumophila*-spiked PBS samples was filter concentrated and treated with the listed concentration of Viability PCR Reagent for 60 min at 37°C. DNA was purified off of filters, and 5 µL of DNA eluate was used as a template using the GoTaq *Legionella* spp./*pneumophila*/SG1 kit as described in Materials and Methods. Quantities of viable *L. pneumophila* in spike samples were also enumerated using Legiolert (green dashed line) or plate-based (blue dashed line) culture-based approaches. Data are expressed in either total genomic unit (GU)/mL (−VR), GU/mL (+VR), most probable number (MPN)/mL (Legiolert), or CFU/mL (plate culture). Error bars represent the standard deviation of triplicate reactions. Images created using www.biorender.com.

A key challenge for any molecular-based bacterial quantitation technique is its ability to produce results that are concordant with accepted culture-based methods. Accordingly, we compared the described *Legionella* viability qPCR workflow to the widely accepted culture-based method, the Legiolert assay from IDEXX (Fig. 4A). To directly compare the two methods, we spiked viable *L. pneumophila* into 200 mL aqueous samples (1× PBS) at three different concentrations—low (~10^1^ CFU/mL), moderate (~10^2^ CFU/mL), and high (~10^3^ CFU/mL)—and processed replicate samples through the respective workflows specified by each method.

The concentration of the sample used in the “high spike” condition was additionally quantified on BCYE plates. Counts determined using the Legiolert assay (9,065 most probable number [MPN]/mL) were approximately fourfold higher than the BCYE plate culture (2,280 CFU/mL) (Fig. 4B), consistent with previous reports of higher quantitation values with the Legiolert (52). Without VR treatment, qPCR values overestimated viable *L. pneumophila* levels by two- to eightfold compared to culture (Fig. 4B), illustrating one of the key difficulties with conventional qPCR. In contrast, when filtered samples were treated with 15–20 µM of Viability PCR Reagent, the resulting number of viable genomic units (~4,500 vGU/mL) of *L. pneumophila* fell between the values obtained with both culture-based techniques (Fig. 4B). Both plate culture and Legiolert were limited to only detecting *L. pneumophila*, but the *L. pneumophila* SG1 and *L.* spp. targets in the qPCR assay exhibited similar quantitative results to those observed for the *L. pneumophila* target (Fig. 4B). The “moderate” and “low” spike replicate experiments demonstrated good agreement between the values determined by viability qPCR and Legiolert (Table 1).

**TABLE 1:** Comparison of vPCR to culture^*a*^

Spike level	Sample	Log vGU/mL	RSD% (vGU/mL)	Log MPN/mL	RSD% (MPN/mL)	Correlation coefficient	RSQ	IA
Moderate	1	2.39 ± 0.02	1.26	2.81 ± 0.00	0.00	0.94	0.88	0.83
	2	1.97 ± 0.07	7.34	2.75 ± 0.04	2.04			
	3	2.17 ± 0.01	1.27	2.41 ± 0.03	2.00			
	4	1.09 ± 0.02	2.94	1.35 ± 0.02	0.00			
Blank	5	N.D.	N.D.	N.D.	N.D.			
Low	6	1.26 ± 0.06	9.16	2.00 ± 0.04	2.12	0.95	0.90	0.82
	7	1.28 ± 0.06	9.17	1.71 ± 0.13	10.82			
	8	0.68 ± 0.05	15.67	1.23 ± 0.04	4.67			
	9	0.33 ± 0.10	40.19	0.04 ± 0.00	0.00			
Blank	10	N.D.	N.D.	N.D.	N.D.			

^*a*^ 200 mL water samples were spiked with either moderate (~10^2^ bacteria/mL, samples 1–4) or low (~10^1^ bacteria/mL, samples 6–9) concentrations of *L. pneumophila* and analyzed using Legiolert (IDEXX) or *Legionella* vPCR workflow (15 µM VR concentration). Sample technical replicate (*n* = 3) values were averaged and log-transformed, and standard error and relative standard deviation were calculated for each sample set. Correlation coefficient, R squared coefficient of determination (RSQ), and index of agreement (IA) were used to compare vGU/mL (vPCR) and MPN/mL (Legiolert). Samples #5 and #10 were the method blanks and were omitted from statistical analyses. N.D., not detected.

### *Legionella* detection in environmental samples

*Legionella* often proliferates in cooling towers (CT), which are highly heterogeneous environments containing diverse abiotic conditions and a broad spectrum of microbial communities (53, 54). Therefore, it was important to examine whether concordance between the viability qPCR and Legiolert methods could be replicated in real-world samples. We sourced CT samples from various locations across New Brunswick, Canada, including hospitals, arenas, and manufacturing facilities. From May to September 2024, 81 CT samples were collected and processed using both the vPCR and Legiolert workflows. To streamline processing, we introduced a freezing step at −20°C following VR neutralization, allowing samples to be stored and batched for extraction. To verify if there was no deleterious effect on sample integrity from the freezing process, we analyzed paired duplicate samples following freezing for a period of 1 month and compared them to those freshly processed and extracted without any freezing. No difference in calculated viable genomic unit/mL was observed between the frozen and freshly purified samples (Table S5).

**Fig 5:**
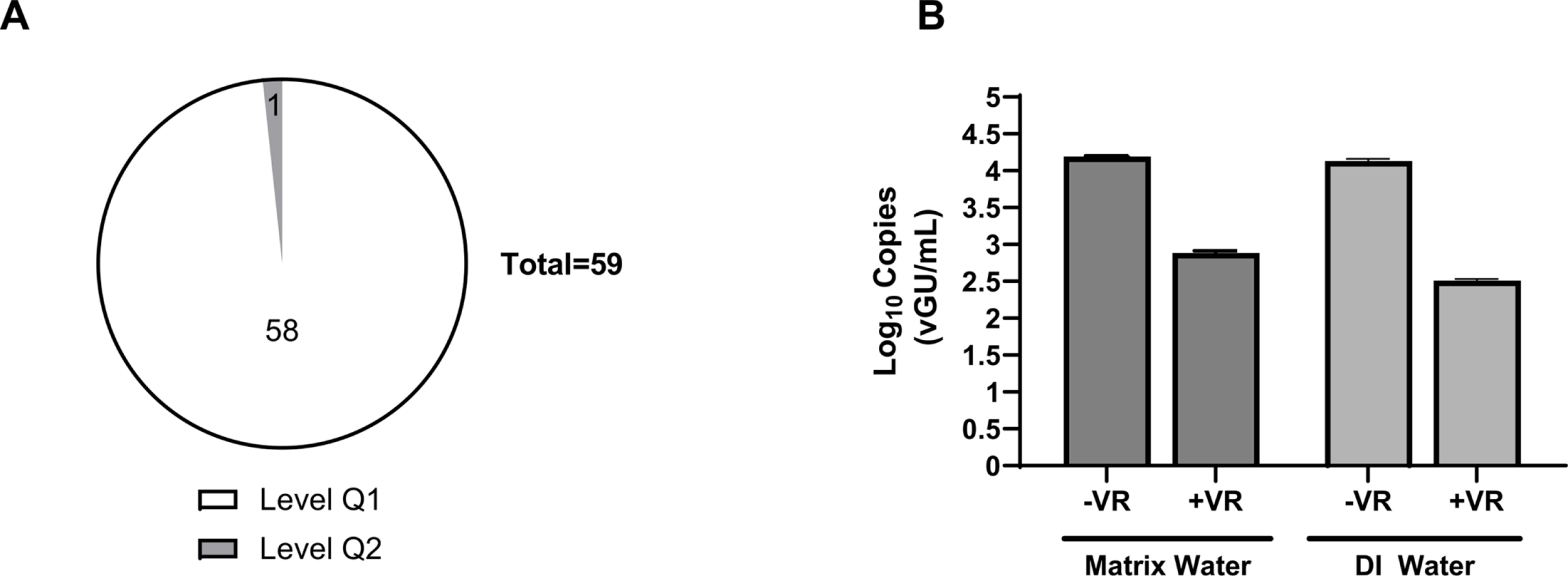
Cooling tower water sampling for *Legionella*. Cooling tower samples (250 mL) were analyzed concurrently by the GoTaq *Legionella* spp./*pneumophila*/SG1 qPCR Kit with Viability PCR Reagent pretreatment (Promega) and Legiolert assay (IDEXX). (**A**) Overview of distribution of samples tested which were below (≤10 vGU/mL, Q1) or above (≥10 vGU/mL) federal guidelines. (**B**) Matrix inhibition testing. Cooling tower water samples (matrix water) and deionized water samples (DI) were spiked with known amounts of *Legionella pneumophila* (spike level ~450 MPN/mL) either treated with 10 µM Viability PCR Reagent (+VR) or not (−VR), nucleic acid purified, and vGU/mL determined as described in Materials and Methods. Data represented as mean ± standard deviation from two independent replicates.

Of the 81 CT samples collected and treated with the viability workflow, 59 were extracted and analyzed via qPCR. Analysis was discontinued after discovering that most samples (96.6%) were negative by Legiolert, with reported values of <1 MPN/mL. The qPCR method demonstrated excellent NPV (NPV: 0.98), as all samples negative by vPCR were also negative by Legiolert. From the public health perspective (55), 98.3% of CT samples tested by both methods were in agreement and fell within acceptable limits (i.e., level Q1, when the *L. pneumophila* bacteria count was less than 10 GU/mL). Only 1.7% of samples were at level Q2 (≤10 GU/mL ≤100), and none reached level Q3 (≥100 GU/mL) (Fig. 5A).

To assess potential matrix inhibition, cooling tower water (MW) and deionized water samples (DW) were spiked with a known amount of *L. pneumophila* (spike level ~450 MPN/mL) ± VR. The cycle threshold (C_t_) value obtained for the samples was then calculated, considering the dilution factors and sample volume used, and expressed as log genomic units/mL. No matrix inhibition effect was observed, as MW spike samples showed similar genomic units/mL values to DW spike samples treated with and without VR, respectively (Fig. 5B). However, the MW spiked samples treated with VR did not exhibit concordance with Legiolert.

## DISCUSSION

Accurately distinguishing viable from non-viable bacteria remains a fundamental challenge in microbiology, with significant implications for diagnostics, public health, and environmental monitoring. Conventional PCR-based assays, while fast and sensitive, cannot differentiate DNA from viable versus non-viable cells, often resulting in overestimation of microbial loads. vPCR addresses this limitation by selectively suppressing amplification of DNA from non-viable organisms. However, existing vPCR reagents such as PMA and EMA require photoactivation, introducing complexity, susceptibility to matrix interference, and limitations in automation and throughput (31–33).

Here, we describe the optimization of the first photoactivation-free vPCR reagent, a compound that irreversibly modifies nucleic acids from non-viable bacteria while being excluded from intact, viable cells. The reagent eliminates the need for light exposure, possibly improving compatibility with turbid or high-organic content matrices and enabling seamless integration into automated sample processing workflows, although both of these advantages need to be directly tested. Its performance was validated across both Gram-positive and Gram-negative organisms, including *L. innocua*, *P. aeruginosa*, and *L. pneumophila*. Optimization of reagent concentration, amplicon length, and treatment format resulted in robust viability discrimination with significant reduction in qPCR signal from non-viable cells.

We further demonstrated the utility of VR in detecting viable *Legionella* from water samples, a relevant public health application. Using a triplex qPCR assay targeting *Legionella* spp., *L. pneumophila*, and *L. pneumophila* SG1, we quantified viability reductions following heat treatment and adapted the workflow for use on filter-concentrated samples. In addition, the results obtained with the VR workflow were concordant with culture-based methods commonly used for *L. pneumophila* detection. Concordance with standard culture-based methods will be a key milestone for any further applications of the VR.

These capabilities address critical gaps in current *Legionella* monitoring, which relies on culture-based methods that require 7–10 days and fail to detect VBNC cells. In contrast, the described vPCR workflow yields results in under 6 hs and enables high-resolution quantification of viable cells, even in complex sample matrices.

The implications of this technology extend well beyond *Legionella* surveillance. This reagent lays the foundation for next-generation viability-based nucleic acid diagnostics applicable to food safety (56), clinical microbiology, bioprocess monitoring, and microbial ecology (57). It provides a potential tool for tracking antimicrobial efficacy (58), measuring disinfection outcomes, and detecting VBNC pathogens that escape both culture and PMA-based detection. Its compatibility with standard qPCR platforms and multiplex assays makes it accessible for widespread adoption. Recent publications have expanded the scope of the VR with utility demonstrated in bacterial biofilms (59) and DNA and RNA viruses (60, 61). Viral detection applications where a straightforward culture system does not exist, such as human norovirus (62) or hepatitis C (63), stand to gain the most from vPCR-based approaches.

Despite the advantages listed above, viability PCR does have limitations that will need to be addressed before widespread implementation of the technology can take place. Standardization is needed, as PCR amplicon lengths, PCR quantitation methodology, sample matrix, and bacterial/viral structure can significantly affect the efficacy of the vPCR approach. We appreciate that the *Legionella* cooling tower biome examined in this report, although presenting a promising environmental application, does not guarantee method applicability in all situations. Environmental samples, in particular, are composed of diverse bacteria with a wide range of cell envelope organizations and stress responses and reside in matrices that are often at the extreme ranges of salinity, turbidity, or total biomass. These factors all potentially alter the functionality of VR, and more investigation is needed into this topic in a variety of environmental microcosms.

In addition, vPCR does not provide a culture isolate for further investigation. The primary advantage of culture-based methods in public health is the ability to produce isolates that enable epidemiological investigations linking clinical cases to environmental reservoirs (64). Future work with vPCR reagent pretreatment prior to next-generation sequencing may help bridge this gap by providing molecular subtyping information specifically from the viable fraction of the microbial genomes in a sample. Additional research is also needed to establish alert levels that translate quantitative molecular results into actionable public health guidance.

In summary, this work evaluates a new class of viability-selective reagent that simplifies and improves molecular quantification of viable bacteria. By eliminating the need for photoactivation and supporting rapid, sequence-specific detection of viable microbes, the VR offers a meaningful advancement for microbiologists seeking faster, more accurate, and operationally simple viability assays. As applications of microbial viability detection continue to expand, tools like this will be instrumental in accelerating both research and public health response efforts.

## MATERIALS AND METHODS

### Strains, oligos, and reagents

All oligonucleotide primers and probes used for qPCR experiments were purchased from IDT (Coralville, IA, USA) or LGC Biosearch (Petaluma, CA, USA) and are listed in Table S1. All bacterial strains are listed in Table S2 in the supplementary material.

### Bacterial growth conditions

For the bacterial optimization experiments, *P. aeruginosa* were grown in lysogeny broth (casein peptone at 10 g/L, Bacto yeast extract at 5 g/L, and sodium chloride at 5 g/L with or without 1.5% [wt/vol] agar). *L. innocua* was grown in terrific broth (Bacto tryptone at 12 g/L, Bacto yeast extract at 24 g/L, glycerol at 0.4% wt/vol, and potassium phosphate at 10% vol/vol with or without 1.5% [wt/vol] agar). *L. pneumophila* was grown in *Legionella* Enrichment Broth Base (Sigma-Aldrich, Burlington, MA, USA) supplemented with Legionella Growth Supplement (Sigma-Aldrich), ACES Buffer at 10 g/L, and alpha-ketoglutarate at 1 g/L.

All bacterial strains were streaked onto solid agar medium listed above and incubated at 37°C until visible colonies were present. Starter cultures were inoculated into liquid broth media and were incubated at 37°C with shaking (200 RPM) until turbid. For Viability PCR Reagent optimization experiments, turbid bacterial cultures were subcultured 1:100 into 50 mL of fresh liquid media and incubated at 37°C with shaking until the exponential phase (OD_600_: 0.2–0.8), upon which cultures were harvested for further experimentation.

For the *Legionella* PBS-spiking experiments, *L. pneumophila* from Culti-Loops (Thermo Scientific, Waltham, MA, USA) were streaked onto BCYE plates (Hardy Diagnostics, Santa Maria, CA, USA) and incubated at 35°C until visible colonies were present. Following incubation, one colony was picked and suspended in 10 mL of 1× PBS (VWR, Radnor, PA, USA) and mixed via vortex mixer. This suspension was serially diluted, with 1 mL of each dilution level inoculated into 100 mL of Legiolert media, with each dilution level performed in triplicate. Following inoculation, the triplicate Legiolert spikes were each mixed via manual inversion and poured into a Quanti-Tray. Quanti-Trays were inspected for air bubbles prior to sealing and then incubated at 37°C for 7 days as per the manufacturer’s instructions (IDEXX Laboratories, Inc., Westbrook, ME, USA). The prepared dilutions were stored at 4°C until the Legiolert data were obtained.

Based on the most probable number/mL concentration, appropriate dilutions were selected for the experiment (~10^1^, ~10^2^, and ~10^3^ most probable number/mL for low, medium, and high spikes, respectively). Three replicates of each spike were then subjected to the viability workflow to compare the viable genomic units/mL with the most probable number/mL obtained from Legiolert for each concentration.

### Bacterial Viability PCR Reagent optimization

Forty milliliters of exponential phase bacterial culture was centrifuged (1,800 × *g*, 10 min) to pellet bacteria, and the supernatant was discarded. Bacterial pellets were resuspended in a volume of 1× PBS to a theoretical OD_600_ of 1.0 (example: pellet from 40 mL of OD_600_: 0.25 culture resuspended in 10 mL 1× PBS). For all bacteria tested, OD_600_ of 1.0 roughly correlates to 10^8^ CFU/mL. Bacterial concentrations in all experiments were verified by culture plating.

From the bacterial culture suspension, 200 μL was aliquoted into two paired 1.5 mL Eppendorf tubes. One tube was incubated at 95°C for 15 min to heat kill (non-viable) the bacterial cells. Following heat treatment, culture suspensions were cooled on ice. The non-heat-killed (viable, no treatment) bacterial aliquot was stored on ice during heat treatment.

Following heating, the bacterial suspensions were returned to room temperature, and the indicated concentration of Viability PCR Reagent (Promega Corporation, Madison, WI, USA) (1–30 µM) was added to both tubes of paired bacterial culture. Tubes were vortexed to mix and incubated statically in the dark for 30 min at 37°C. Tubes were revortexed to mix, followed by an additional 30-min static incubation at 37°C. A 20 µL volume of 10× neutralization buffer was added to each tube and vortexed to mix. After a 15-min static incubation at room temperature, bacterial nucleic acid was purified using a Maxwell RSC automated purification instrument (Promega Corporation, Madison, WI, USA) in conjunction with the Maxwell RSC PureWater Kit (Promega Corporation, Madison, WI, USA), eluting in a final volume of 80 µL. Nucleic acid was stored at −20°C until further analysis.

For the PMAxx comparison, 10 µM of PMAxx (Biotium, Fremont, CA, USA) was added to the viable and non-viable cell suspensions, incubated in the dark at 37°C for 30 min, followed by a 30-min blue light exposure using the PMA-Lite LED photolysis device (Biotium). Bacterial nucleic acid was purified using a Maxwell RSC automated purification instrument in conjunction with the Maxwell RSC PureWater Kit (Promega Corporation), eluting in a final volume of 80 µL. Nucleic acid was stored at −20°C until further analysis.

### qPCR amplification and nucleic acid quantification

For viability qPCR experiments using *L. innocua* and *P. aeruginosa*, eluted nucleic acid was used to perform dye-based qPCR using the GoTaq qPCR System (Promega Corporation). qPCR reactions targeted amplicons specific to each bacterium, as outlined in Table S1. The 20 µL amplification reactions were composed of 18 µL reaction master mix and 2 µL nucleic acid with 2 µL of nuclease-free water used in place of nucleic acid for a no-template control. PCR reactions were performed on a Bio-Rad CFX96 Thermocycler (Bio-Rad, Hercules, CA, USA) with the following cycling conditions: 3 min at 95°C and 40 cycles of 45 s at 95°C, 60 s at 58°C, and 60 s at 72°C. After PCR cycling, the following melt curve procedure was performed to ensure qPCR specificity: stepwise gradient (0.5°C per step, 5 s per step with fluorescent read after each step) from 65°C to 95°C. qPCR reactions were run in triplicate. The ΔC_t_ was defined as the difference in C_t_ value between the non-viable (heat killed) and viable cells (ΔC_t_ = C_t-Non-viable_ − C_t-Viable_).

For viability qPCR experiments using *L. pneumophila*, eluted nucleic acid was used to perform probe-hydrolysis qPCR using the GoTaq *Legionella* spp./*pneumophila*/SG1 qPCR Kit (Promega Corporation) using genus, species, and serogroup specific primers and probes (Table S1). The multiplex qPCR assay simultaneously detects and quantifies *L*egionella spp., *L. pneumophila*, and *L. pneumophila* SG1 in a specific, linear (*R*^2^ > 0.97), and efficient manner (90% ≤ E ≤ 110%) (Fig S2; Tables S3 and S4). A linearized plasmid template containing a 435 bp *luc* sequence is included in the primer-probe mix and acts as an IPC. Twenty microliter amplification reactions were composed of 15 µL reaction master mix and 5 µL nucleic acid (eluted sample, bacterial genomic DNA, or quantification standard). Five microliters of nuclease-free water was used as a no-template control. PCR reactions were performed on either a QuantStudio 5 Real-Time Thermocycler (Applied Biosystems, Waltham, MA, USA) or a CFX96 Real-Time Thermocycler (Bio-Rad) with the following cycling conditions: 2 min at 95°C, then 40 cycles of 15 s at 95°C and 60 s at 61°C. All qPCR reactions were run in triplicate. The ΔC_t_ was defined as the difference in C_t_ value between the non-viable (heat killed) and viable cells (ΔC_t_ = C_t-Non-viable_ − C_t-Viable_).

The *Legionella* Quant Standard (included in the GoTaq *Legionella* spp./*pneumophila*/SG1 qPCR Kit) was used to create 5-log standard curves (20–200,000 copies/well) in the *Legionella* qPCR reactions described above. This standard curve was used to quantify the concentration of *Legionella* spp., *L. pneumophila*, and *L. pneumophila* SG1 genomic DNA in each eluted sample.

### *Legionella* qPCR assay specificity testing

Genomic DNA from 65 bacterial isolates (37 *Legionella* and 28 non-*Legionella*, Tables S3 and S4) was tested as PCR template for the GoTaq *Legionella* spp./*pneumophila*/SG1 qPCR Kit. Inclusivity tests were performed at a DNA concentration of 100 GU/well. Exclusivity tests were performed at a DNA concentration of 10,000 GU/well. The qPCR amplification reactions were performed as described above. A reaction was considered positive for amplification if C_t_/C_q_ is <40. IPC amplification was included to ensure qPCR reactions were proficient for nucleic acid amplification.

### On-filter Viability PCR Reagent workflow optimization

A 200 mL suspension of 10^3^ bacteria/mL non-viable (heat-killed) *L. pneumophila* in 1× PBS was filter concentrated onto 25 mm diameter polycarbonate filters with a 0.2 µm pore size (Sigma-Aldrich) using a multi-adaptor vacuum manifold (Cytiva, Marlborough, MA, USA). Following filtration, filters were placed in a sterile petri dish and cut into two pieces using a sterile scalpel. Filter fragments for each treatment were transferred to tubes containing 700 µL of 1× PBS. The indicated concentration of Viability PCR Reagent (10 or 20 µM) was added to each tube. Tubes were vortexed to mix and incubated statically in the dark for 30 min at 37°C. Tubes were revortexed to mix, followed by an additional 30-min static incubation at 37°C. To each tube, 20 µL of 10× neutralization buffer was added, vortexing to mix. After a 15-min static incubation at room temperature, bacterial nucleic acid was purified using a Maxwell RSC automated purification instrument in conjunction with the Maxwell RSC PureWater Kit (Promega Corporation), following the manufacturer’s instructions, eluting in a final volume of 80 µL. Nucleic acid was stored at −20°C until further analysis.

### *Legionella* evaluation study

The 200 mL suspensions of *L. pneumophila* at low (10^1^ bacteria/200 mL), moderate (10^2^ bacteria/200 mL), and high (10^3^ bacteria/200 mL) concentrations were prepared, with four (low and moderate) or two (high) biological replicates per sample concentration. Each 200 mL suspension was passed through the filtration apparatus, whereafter filter papers with concentrated samples were sheared and placed into 700 µL of 1× PBS (VWR). Suspended filter papers were then treated with diluted VR (4,500 µM) to a final concentration of 5–30 µM VR per suspension (15 µM for moderate and low spikes). Samples were then mixed briefly by vortexing. Following the mixing step, samples were allowed to incubate at 37°C for 60 min with a brief pause to revortex samples after 30 min. The VR in the samples was neutralized using 70 µL of 10× neutralization buffer and allowed to incubate at room temperature for an additional 15 min. Samples were then stored frozen at −20°C prior to DNA extraction and purification. Nucleic acid purification was performed using the DNeasy PowerWater Kit (Qiagen, Hilden, Germany) as per the manufacturer’s instructions, eluting to a final volume of 50 µL.

Following DNA purification, the GoTaq *Legionella* spp./*pneumophila*/SG1 qPCR Kit (Promega Corporation) was used to quantify viable genomic units (vGU/mL) in the original spiked sample. The results obtained were compared to those produced by the Legiolert assay (IDEXX Laboratories, Inc.) used to assess the most probable number (MPN/mL) of viable cells in the original spiked sample.

### Environmental sampling

Eighty-one water samples were collected from cooling towers located in factories, commercial buildings, and hospitals across New Brunswick (NB) between 16 May and 4 October 2024. CT samples were from both surface and groundwater sources collected by the clients in sterilized 250 mL HDPE Tamper Evident Bottles (Systems Plus, Baden, ON), and shipped to the testing laboratory (RPC, Moncton, NB) on wet ice. Due to client confidentiality, samples were not identified to a particular cooling tower. The scope of this study was to compare the two methods; therefore, no water characteristics were recorded at the time of water collection. Samples were then stored at the laboratory from 1°C to 7°C until analysis (within 48 h of sample collection). The presence of *L. pneumophila* was detected using the IDEXX Legiolert methods as laboratory gold standard. Legiolert was evaluated using a non-potable protocol (Legiolert-1 mL; the sensitivity of Legiolert-1 mL is sufficient for testing to the guideline value for cooling tower water). Briefly, for the Legiolert-1 mL test, 2 mL of pretreatment reagent (Legiolert Pretreatment; IDEXX Laboratories, Inc.) was added to 2 mL of cooling tower water. After incubating the mixture at room temperature for 1 min, 2 mL of the pretreated sample was transferred into 100 mL of Legiolert liquid medium (prepared by dissolving one packet of Legiolert powder in 100 mL of sterile water). The final mixture was then dispensed into a Quanti-Tray/Legiolert and sealed using the IDEXX Sealer Plus. Samples were incubated at 37°C ± 0.5°C for 7 days. Following incubation, wells exhibiting a brown color change or turbidity were counted as positive, and the concentration of *L. pneumophila* was determined using the Legiolert MPN table.

Samples were also analyzed concurrently by the GoTaq *Legionella* spp./*pneumophila*/SG1 qPCR Kit (15 µM VR concentration, Promega Corporation) and Legiolert assay (IDEXX Laboratories, Inc.).

### Statistical analyses

Statistical comparisons were performed with GraphPad Prism (v10.1.2; Dotmatics, Boston, MA, USA). Two-tailed unpaired *t*-tests and ordinary one-way ANOVAs were performed for noted comparisons in the manuscript.

Relative standard deviation (RSD%) was calculated as follows:


$$RSD\left(\%\right)=\frac{\sigma }{\mu }\;\times \;100,$$

where *σ* is the sample standard deviation and *μ* is the sample mean.

## Data Availability

Raw data for all figures are uploaded to Open Science Framework at the following link: https://osf.io/md5ws/?view_only=26a38d70fb034772ac1f1e50af04e932.

## SUPPLEMENTAL MATERIAL

Supplemental material spectrum.03935-25-s0001.docx Fig. S1 and S2; Tables S1 to S5.

ASM does not own the copyrights to Supplemental Material that may be linked to, or accessed through, an article. The authors have granted ASM a non-exclusive, world-wide license to publish the Supplemental Material files. Please contact the corresponding author directly for reuse.

## References

[1] Bogosian G, Bourneuf EV. 2001. A matter of bacterial life and death. EMBO Rep 2:770–774. doi:10.1093/embo-reports/kve182 11559589 PMC1084037

[2] Xu HS, Roberts N, Singleton FL, Attwell RW, Grimes DJ, Colwell RR. 1982. Survival and viability of nonculturable Escherichia coli and Vibrio cholerae in the estuarine and marine environment. Microb Ecol 8:313–323. doi:10.1007/BF02010671 24226049

[3] Li L, Mendis N, Trigui H, Oliver JD, Faucher SP. 2014. The importance of the viable but non-culturable state in human bacterial pathogens. Front Microbiol 5:258. doi:10.3389/fmicb.2014.00258 24917854 PMC4040921

[4] Oliver JD. 2005. The viable but nonculturable state in bacteria. J Microbiol 43 Spec No:93–100. 15765062

[5] Emerson JB, Adams RI, Román CMB, Brooks B, Coil DA, Dahlhausen K, Ganz HH, Hartmann EM, Hsu T, Justice NB, Paulino-Lima IG, Luongo JC, Lymperopoulou DS, Gomez-Silvan C, Rothschild-Mancinelli B, Balk M, Huttenhower C, Nocker A, Vaishampayan P, Rothschild LJ. 2017. Schrödinger’s microbes: tools for distinguishing the living from the dead in microbial ecosystems. Microbiome 5:86. doi:10.1186/s40168-017-0285-3 28810907 PMC5558654

[6] Davey HM. 2011. Life, death, and in-between: meanings and methods in microbiology. Appl Environ Microbiol 77:5571–5576. doi:10.1128/AEM.00744-11 21705550 PMC3165249

[7] Jung JH, Lee JE. 2016. Real-time bacterial microcolony counting using on-chip microscopy. Sci Rep 6:21473. doi:10.1038/srep21473 26902822 PMC4763285

[8] Boye E, Løbner-Olesen A. 1991. Bacterial growth control studied by flow cytometry. Res Microbiol 142:131–135. doi:10.1016/0923-2508(91)90020-b 1925010

[9] Venkateswaran K, Hattori N, La Duc MT, Kern R. 2003. ATP as a biomarker of viable microorganisms in clean-room facilities. J Microbiol Methods 52:367–377. doi:10.1016/s0167-7012(02)00192-6 12531506

[10] Blazewicz SJ, Barnard RL, Daly RA, Firestone MK. 2013. Evaluating rRNA as an indicator of microbial activity in environmental communities: limitations and uses. ISME J 7:2061–2068. doi:10.1038/ismej.2013.102 23823491 PMC3806256

[11] Nisar MA, Ross KE, Brown MH, Bentham R, Best G, Whiley H. 2023. Detection and quantification of viable but non-culturable Legionella pneumophila from water samples using flow cytometry-cell sorting and quantitative PCR. Front Microbiol 14:1094877. doi:10.3389/fmicb.2023.1094877 36793878 PMC9922708

[12] Josephson KL, Gerba CP, Pepper IL. 1993. Polymerase chain reaction detection of nonviable bacterial pathogens. Appl Environ Microbiol 59:3513–3515. doi:10.1128/aem.59.10.3513-3515.1993 8250575 PMC182487

[13] López-Saucedo C, Cerna JF, Villegas-Sepulveda N, Thompson R, Velazquez FR, Torres J, Tarr PI, Estrada-García T. 2003. Single multiplex polymerase chain reaction to detect diverse loci associated with diarrheagenic Escherichia coli. Emerg Infect Dis 9:127–131. doi:10.3201/eid0901.010507 12533296 PMC2873745

[14] Li B, Liu H, Wang W. 2017. Multiplex real-time PCR assay for detection of Escherichia coli O157:H7 and screening for non-O157 Shiga toxin-producing E. coli. BMC Microbiol 17:215. doi:10.1186/s12866-017-1123-2 29121863 PMC5679507

[15] Whiley H, Taylor M. 2016. Legionella detection by culture and qPCR: comparing apples and oranges. Crit Rev Microbiol 42:65–74. doi:10.3109/1040841X.2014.885930 24580080

[16] Fittipaldi M, Nocker A, Codony F. 2012. Progress in understanding preferential detection of live cells using viability dyes in combination with DNA amplification. J Microbiol Methods 91:276–289. doi:10.1016/j.mimet.2012.08.007 22940102

[17] Nocker A, Camper AK. 2009. Novel approaches toward preferential detection of viable cells using nucleic acid amplification techniques. FEMS Microbiol Lett 291:137–142. doi:10.1111/j.1574-6968.2008.01429.x 19054073

[18] Nocker A, Sossa KE, Camper AK. 2007. Molecular monitoring of disinfection efficacy using propidium monoazide in combination with quantitative PCR. J Microbiol Methods 70:252–260. doi:10.1016/j.mimet.2007.04.014 17544161

[19] Venkobachar C, Iyengar L, Prabhakara Rao AVS. 1977. Mechanism of disinfection: effect of chlorine on cell membrane functions. Water Res 11:727–729. doi:10.1016/0043-1354(77)90114-2

[20] Villarino A, Bouvet OM, Regnault B, Martin-Delautre S, Grimont PAD. 2000. Exploring the frontier between life and death in Escherichia coli: evaluation of different viability markers in live and heat- or UV-killed cells. Res Microbiol 151:755–768. doi:10.1016/s0923-2508(00)01141-4 11130866

[21] Nogva HK, Drømtorp SM, Nissen H, Rudi K. 2003. Ethidium monoazide for DNA-based differentiation of viable and dead bacteria by 5’-nuclease PCR. Biotechniques 34:804–808, doi:10.2144/03344rr02 12703305

[22] Nocker A, Cheung CY, Camper AK. 2006. Comparison of propidium monoazide with ethidium monoazide for differentiation of live vs. dead bacteria by selective removal of DNA from dead cells. J Microbiol Methods 67:310–320. doi:10.1016/j.mimet.2006.04.015 16753236

[23] Hixon SC, White WE, Yielding KL. 1975. Selective covalent binding of an ethidium analog to mitochondrial DNA with production of petite mutants in yeast by photoaffinity labelling. J Mol Biol 92:319–329. doi:10.1016/0022-2836(75)90231-4 1095756

[24] Rudi K, Moen B, Drømtorp SM, Holck AL. 2005. Use of ethidium monoazide and PCR in combination for quantification of viable and dead cells in complex samples. Appl Environ Microbiol 71:1018–1024. doi:10.1128/AEM.71.2.1018-1024.2005 15691961 PMC546808

[25] Soejima T, Iida K, Qin T, Taniai H, Seki M, Takade A, Yoshida S. 2007. Photoactivated ethidium monoazide directly cleaves bacterial DNA and is applied to PCR for discrimination of live and dead bacteria. Microbiol Immunol 51:763–775. doi:10.1111/j.1348-0421.2007.tb03966.x 17704639

[26] Lee JL, Levin RE. 2009. A comparative study of the ability of EMA and PMA to distinguish viable from heat killed mixed bacterial flora from fish fillets. J Microbiol Methods 76:93–96. doi:10.1016/j.mimet.2008.08.008 18817818

[27] Chen NT, Chang CW. 2010. Rapid quantification of viable legionellae in water and biofilm using ethidium monoazide coupled with real-time quantitative PCR. J Appl Microbiol 109:623–634. doi:10.1111/j.1365-2672.2010.04678.x 20163500

[28] Scaturro M, Fontana S, Dell’eva I, Helfer F, Marchio M, Stefanetti MV, Cavallaro M, Miglietta M, Montagna MT, De Giglio O, et al.. 2016. A multicenter study of viable PCR using propidium monoazide to detect Legionella in water samples. Diagn Microbiol Infect Dis 85:283–288. doi:10.1016/j.diagmicrobio.2016.04.009 27133308

[29] Son H-S, Yun K-W, Seong M-J, Lee S-M, Kim M-C. 2026. Propidium monoazide-quantitative PCR for antibiotic sensitivity testing and minimum inhibitory concentration testing of antibiotic-resistant bacteria. Water Biol Secur 5:100406. doi:10.1016/j.watbs.2025.100406

[30] Nocker A, Camper AK. 2006. Selective removal of DNA from dead cells of mixed bacterial communities by use of ethidium monoazide. Appl Environ Microbiol 72:1997–2004. doi:10.1128/AEM.72.3.1997-2004.2006 16517648 PMC1393219

[31] Flekna G, Stefanic P, Wagner M, Smulders FJM, Mozina SS, Hein I. 2007. Insufficient differentiation of live and dead Campylobacter jejuni and Listeria monocytogenes cells by ethidium monoazide (EMA) compromises EMA/real-time PCR. Res Microbiol 158:405–412. doi:10.1016/j.resmic.2007.02.008 17449228

[32] Kobayashi H, Oethinger M, Tuohy MJ, Hall GS, Bauer TW. 2009. Unsuitable distinction between viable and dead Staphylococcus aureus and Staphylococcus epidermidis by ethidium bromide monoazide. Lett Appl Microbiol 48:633–638. doi:10.1111/j.1472-765X.2009.02585.x 19416465

[33] Kaur S, Bran L, Rudakov G, Wang J, Verma MS. 2025. Propidium monoazide is unreliable for quantitative live-dead molecular assays. Anal Chem 97:2914–2921. doi:10.1021/acs.analchem.4c05593 39870608 PMC11822742

[34] Fittipaldi M, Codony F, Adrados B, Camper AK, Morató J. 2011. Viable real-time PCR in environmental samples: can all data be interpreted directly? Microb Ecol 61:7–12. doi:10.1007/s00248-010-9719-1 20632000

[35] Varma M, Field R, Stinson M, Rukovets B, Wymer L, Haugland R. 2009. Quantitative real-time PCR analysis of total and propidium monoazide-resistant fecal indicator bacteria in wastewater. Water Res 43:4790–4801. doi:10.1016/j.watres.2009.05.031 19540546

[36] Bae S, Wuertz S. 2009. Discrimination of viable and dead fecal Bacteroidales bacteria by quantitative PCR with propidium monoazide. Appl Environ Microbiol 75:2940–2944. doi:10.1128/AEM.01333-08 19270114 PMC2681701

[37] Russell AD, Harries D. 1967. Some aspects of thermal injury in Escherichia coli. Appl Microbiol 15:407–410. doi:10.1128/am.15.2.407-410.1967 5339842 PMC546913

[38] Contreras PJ, Urrutia H, Sossa K, Nocker A. 2011. Effect of PCR amplicon length on suppressing signals from membrane-compromised cells by propidium monoazide treatment. J Microbiol Methods 87:89–95. doi:10.1016/j.mimet.2011.07.016 21821068

[39] Fields BS, Benson RF, Besser RE. 2002. Legionella and Legionnaires’ disease: 25 years of investigation. Clin Microbiol Rev 15:506–526. doi:10.1128/CMR.15.3.506-526.2002 12097254 PMC118082

[40] Gaia V, Fry NK, Afshar B, Lück PC, Meugnier H, Etienne J, Peduzzi R, Harrison TG. 2005. Consensus sequence-based scheme for epidemiological typing of clinical and environmental isolates of Legionella pneumophila. J Clin Microbiol 43:2047–2052. doi:10.1128/JCM.43.5.2047-2052.2005 15872220 PMC1153775

[41] ESCMID. 2019. Uniting specialists to control Legionnaires’ disease through efficient research, tracking, and diagnosis. Available from: https://www.escmid.org/esgli/

[42] Lucas CE, Taylor TH, Fields BS. 2011. Accuracy and precision of Legionella isolation by US laboratories in the ELITE program pilot study. Water Res 45:4428–4436. doi:10.1016/j.watres.2011.05.030 21726887

[43] Ducret A, Chabalier M, Dukan S. 2014. Characterization and resuscitation of “non-culturable” cells of Legionella pneumophila. BMC Microbiol 14:3. doi:10.1186/1471-2180-14-3 24383402 PMC3882098

[44] Hwang MG, Katayama H, Ohgaki S. 2006. Intracellular survivability of legionella pneumophila in VBNC state against silver and copper exposure. Environ Eng Res 43:237–243. doi: 10.11532/proes1992.43.237

[45] García MT, Jones S, Pelaz C, Millar RD, Abu Kwaik Y. 2007. Acanthamoeba polyphaga resuscitates viable non-culturable Legionella pneumophila after disinfection. Environ Microbiol 9:1267–1277. doi:10.1111/j.1462-2920.2007.01245.x 17472639

[46] Fields BS, Shotts EB, Feeley JC, Gorman GW, Martin WT. 1984. Proliferation of Legionella pneumophila as an intracellular parasite of the ciliated protozoan Tetrahymena pyriformis. Appl Environ Microbiol 47:467–471. doi:10.1128/aem.47.3.467-471.1984 6424568 PMC239703

[47] Díaz-Flores Á, Montero JC, Castro FJ, Alejandres EM, Bayón C, Solís I, Fernández-Lafuente R, Rodríguez G. 2015. Comparing methods of determining Legionella spp. in complex water matrices. BMC Microbiol 15:91. doi:10.1186/s12866-015-0423-7 25925400 PMC4436101

[48] Petzold M, Zacharias N, Uhle S, Kieper L, Mutters NT, Kistemann T, Schreiber C. 2025. PCR-based Legionella risk evaluation of drinking water systems-an empirical field evaluation. Microorganisms 13:1311. doi:10.3390/microorganisms13061311 40572199 PMC12195026

[49] Redwitz J, Streich P, Zamfir M, Walser-Reichenbach SM, Seidel M, Herr CEW, Heinze S, Quartucci C. 2024. Verification and application of qPCR and viability-qPCR for Legionella monitoring in evaporative cooling systems complementing the conventional culture method. Sci Total Environ 953:176011. doi:10.1016/j.scitotenv.2024.176011 39236821

[50] Redwitz J, Chai RCJ, Zamfir M, Walser-Reichenbach SM, Herr CEW, Heinze S, Quartucci C. 2024. Analysis of water and aerosol samples of tunnel car washes operated with recycled water for Legionella with culture, qPCR and viability-qPCR. Sci Total Environ 957:177673. doi:10.1016/j.scitotenv.2024.177673 39571807

[51] Walser SM, Gerstner DG, Brenner B, Höller C, Liebl B, Herr CEW. 2014. Assessing the environmental health relevance of cooling towers--a systematic review of legionellosis outbreaks. Int J Hyg Environ Health 217:145–154. doi:10.1016/j.ijheh.2013.08.002 24100053

[52] Sartory DP, Spies K, Lange B, Schneider S, Langer B. 2017. Evaluation of a most probable number method for the enumeration of Legionella pneumophila from potable and related water samples. Lett Appl Microbiol 64:271–275. doi:10.1111/lam.12719 28117485

[53] Wéry N, Bru-Adan V, Minervini C, Delgénes J-P, Garrelly L, Godon J-J. 2008. Dynamics of Legionella spp. and bacterial populations during the proliferation of L. pneumophila in a cooling tower facility. Appl Environ Microbiol 74:3030–3037. doi:10.1128/AEM.02760-07 18390683 PMC2394956

[54] Yamamoto H, Sugiura M, Kusunoki S, Ezaki T, Ikedo M, Yabuuchi E. 1992. Factors stimulating propagation of legionellae in cooling tower water. Appl Environ Microbiol 58:1394–1397. doi:10.1128/aem.58.4.1394-1397.1992 16348704 PMC195609

[55] Canada PWaGS. 2013. MD15161. Public Works and Government Services Canada, Canada.

[56] Bej AK, DiCesare JL, Haff L, Atlas RM. 1991. Detection of Escherichia coli and Shigella spp. in water by using the polymerase chain reaction and gene probes for uid. Appl Environ Microbiol 57:1013–1017. doi:10.1128/aem.57.4.1013-1017.1991 2059028 PMC182838

[57] Villarreal JV, Schwartz T, Obst U. 2010. Culture-independent techniques applied to food industry water surveillance--a case study. Int J Food Microbiol 141 Suppl 1:S147–55. doi:10.1016/j.ijfoodmicro.2010.03.001 20363042

[58] Lee S, Bae S. 2018. Molecular viability testing of viable but non‐culturable bacteria induced by antibiotic exposure. Microb Biotechnol 11:1008–1016. doi:10.1111/1751-7915.13039 29243404 PMC6196391

[59] Lehmusvaara S, Sillanpää A, Wouters M, Korhonen R, Vahvelainen N, Luukinen H, Deptula P, Savijoki K, Hammarén M, Parikka M. 2025. M.marinum lacking epsH shows increased biofilm formation in vitro and boosted antibiotic tolerance in zebrafish. NPJ Biofilms Microbiomes 11:109. doi:10.1038/s41522-025-00743-5 40517184 PMC12167362

[60] Wales SQ, Pandiscia A, Kulka M, Sanchez G, Randazzo W. 2024. Challenges for estimating human norovirus infectivity by viability RT-qPCR as compared to replication in human intestinal enteroids. Int J Food Microbiol 411:110507. doi:10.1016/j.ijfoodmicro.2023.110507 38043474

[61] Randazzo W, Piqueras J, Rodríguez-Díaz J, Aznar R, Sánchez G. 2018. Improving efficiency of viability-qPCR for selective detection of infectious HAV in food and water samples. J Appl Microbiol 124:958–964. doi:10.1111/jam.13519 28649706

[62] Koo ES, Yoo C-H, Na Y, Park SY, Lyoo HR, Jeong YS. 2012. Reliability of non-culturable virus monitoring by PCR-based detection methods in environmental waters containing various concentrations of target RNA. J Microbiol 50:726–734. doi:10.1007/s12275-012-2279-y 23124739

[63] Lohmann V, Bartenschlager R. 2014. On the history of hepatitis C virus cell culture systems. J Med Chem 57:1627–1642. doi:10.1021/jm401401n 24164647

[64] Kirkup BC. 2013. Culture-independence for surveillance and epidemiology. Pathogens 2:556–570. doi:10.3390/pathogens2030556 25437208 PMC4235693

