# Supplementary material for: A photoactivation-free viability PCR reagent for rapid, culture-independent detection of live bacteria
Source: Microbiol Spectr. 2026 Feb 27;14(4):e03935-25. doi: 10.1128/spectrum.03935-25 (PMC13055399; doi:10.1128/spectrum.03935-25)
Supplement: Supplemental material — Fig. S1 and S2; Tables S1 to S5. [file spectrum.03935-25-s0001.docx]

**A Photoactivation-Free Viability PCR Reagent for Rapid, Culture-Independent Detection of Live Bacteria**

Nathan Feirer^1*^, Matthew Poole^2^, Nishanth Kuchi^2**^, Caitlin Hawthorn^4^, Rebecca Marino^4^, James J. Cali^1^, Attiq Rehman^2,3^, Diane Botelho ^2,3^, Subhanjan Mondal^1^

^1^ Promega Corporation, 5430 East Cheryl Parkway, Fitchburg, WI 53711

^2^ Research and Productivity Council (RPC), 115A Harrisville Blvd, Moncton, NB E1H 3T3, Canada

^3^Research and Productivity Council (RPC), 921 College Hill Rd, Fredericton, NB  E3B 6Z9 Canada

^5^ PACE Analytical, 1401 Forbes Avenue, Pittsburgh, PA 15219

*Corresponding Author

**Current Address: Microbix, 265 Watline Avenue, Mississauga, ON L4Z 1P3

**Supplementary Information**

**B**

**A**


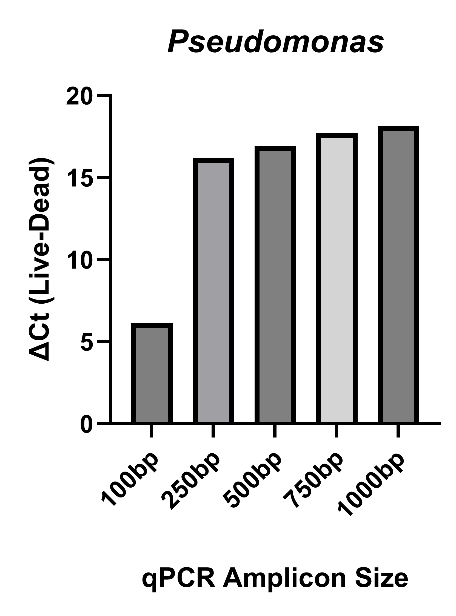

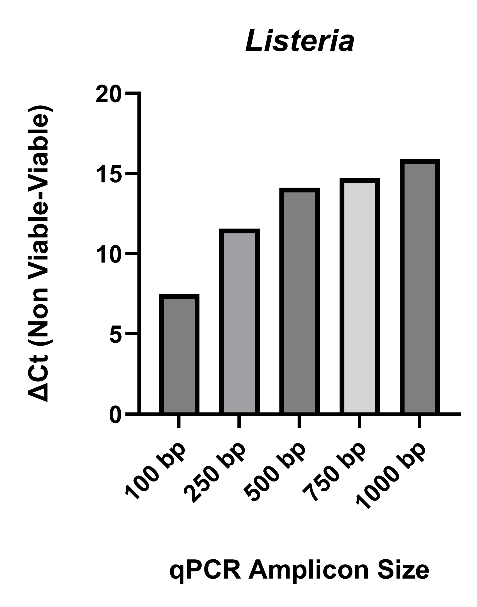


**Figure S1.** Effect of qPCR amplicon length on Viability PCR performance with PMAxx. *Listeria* (A) or *Pseudomonas* (B) (viable and heat-killed) were treated with 10uM of PMAxx, followed by nucleic acid purification. Dye-based qPCR was performed using bacterial specific primers amplifying DNA fragments of the indicated sizes. The ΔC_t_ value is defined as the cycle threshold (C_t_) difference between viable and heat-killed bacterial cells.


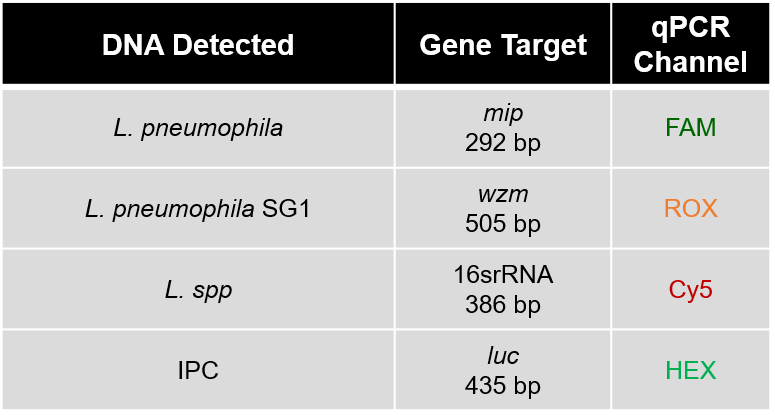


**A**

**B**

**Figure S2.** *Legionella* multiplex qPCR Assay Design. (A) Overview of targets in multiplex qPCR assay used in the manuscript. Primer and probe details are in supplemental information (Table S1) IPC: Internal Positive Control. (B) *Legionella* multiplex assay standard curve. Quantitative Standard DNA containing templates for each *Legionella* qPCR target was diluted to a 5-log range (20-200,00 copies per well) and qPCR assay was performed using the assay described in (A). Efficiency and Linearity (R^2^) values were calculated by QuantStudio Design and Analysis software (Applied Biosystems. Waltham, MA). Error bars represent the standard deviation of four replicate reaction wells.

| **Oligo** | **Microbial Target** | **Sequence** | **Amplicon Size** | **Reference** |
| --- | --- | --- | --- | --- |
| Pa GyrB F | *Pseudomonas aeruginosa* | 5’-GGTGTTCGAGGTGGTGGATA-3’ | See below (R1-R4) | Tavernier and Coenye(1) |
| Pa GyrB R1 | *Pseudomonas aeruginosa* | 5’-TGGTGATGCTGATTTCGCTG-3’ | 71 bp | This Study |
| Pa GyrB R2 | *Pseudomonas aeruginosa* | 5’-TTCAGACCGCCTTCGTACTT-3’ | 549 bp | This Study |
| PaGyrB R3 | *Pseudomonas aeruginosa* | 5’-GTTGTTCAGGTTACGCGTCA -3’ | 769 bp | This Study |
| PaGyrB R4 | *Pseudomonas aeruginosa* | 5’-GATGGTCAGGCGTAGTTCAT-3’ | 286 bp | This Study |
| PaGyrB R5 | *Pseudomonas aeruginosa* | 5’- CCTTGGCTTCGTTGGGATTC -3’ | 992 bp | This Study |
| Lis1B RP | *Listeria innocua* | 5’-TTATACGCGACCGAAGCCAA-3’ | See Below (F1-F4)  93 bp | Bubert *et al.*(2) |
| List F1 | *Listeria innocua* | 5’- CAAATGATTAACGCGCAAGACA -3’ |  | This Study |
| List F2 | *Listeria innocua* | 5’- TGATTGCTCCGGTTTCACTAA -3’ | 274 bp | This Study |
| List F3 | *Listeria innocua* | 5’- CCAACTCAAGCTGCGAAACC -3’ | 516 bp | This Study |
| List F4 | *Listeria innocua* | 5’-GAAGTACGGTGTTTCTGTTCAAG-3’ | 763 bp | This Study |
| Lpneu-276F | *Legionella pneumophila* | 5’-AATTGGTGACTGCAGCTGTT-3’ | 276 bp | This Study |
| Lpneu-276R | *Legionella pneumophila* | 5’-CAGCAGTACGCTTAGCCATC-3’ | 763 bp | This Study |
| Wieland | *Legionella pneumophila* | 5’-TGGTGACTGCAGCTGTTATG-3’ | See Below  633 bp | Kontchou and Nocker(3) |
| Lpm2-R | *Legionella pneumophila* | 5’-GGCCAATAGGTCCGCCAACG-3’ |  | Kontchou and Nocker(3) |
| Lp-Probe 1 | *Legionella pneumophila* | 5’-FAM-CTCATAGCGTCTTGCATGCCT-BHQ1-3’ | N/A | Kontchou and Nocker(3) |
| LpNF-R292 | *Legionella pneumophila* | 5’-GGCCATATGCAAGACCTGAG-3’ | 292 bp | This Study |
| Sg1-P1 | *Legionella pneumophila* SG1 | 5’ TTACCGCTTGCTTTTATGGA 3’ | 505 bp | Merault *et al.*(4) |
| Sg1-P3-NF505 | *Legionella pneumophila* SG1 | 5’ GCCAGAGGCATAAAATGTTTAATATC 3’ | 292 bp | This Study |
| sg1-pb-ROX | *Legionella pneumophila* SG1 | 5’-56ROX-TCTTGGGATTGGGTTGGGTTATTTTAACTCCT-3BHQ_2-3’ | N/A  386 bp | Merault *et al.*(4) |
| JFP | *L. spp* | 5’-AGGGTTGATAGGTTAAGAGC-3’ |  | Wellinghausen *et al*.(5) |
| JRP | *L. spp* | 5’-CCAACAGCTAGTTGACATCG-3’ | N/A | Wellinghausen *et al*.(5) |
| Leg. Gen Probe-2-Quas | *L. spp* | 5'-Quasar 670-AGTGGCGAAGGCGGCTACCT-BHQ2-3' | N/A  435 bp | Wellinghausen *et al*.(5) |
| CC169 | IPC | 5’-CATTCCGGATACTGCGATTTTAAGTG-3’ |  | This Study |
| CC224 | IPC | 5’-AGATCCACAACCTTCGCTTCAAA-3’ | N/A | This Study |
| DS121-Org-560 | IPC | 5'-CalFluor Orange 560 CGCCCCCAGAAGCAATTTCGTGTAAA-BHQ1-3' | N/A | This Study |
|  |  |  |  |  |
|  |  |  |  |  |

**Table S1**: Primers and probes used in this study. Oligos were ordered from IDT (Coralville, IA) or LGC Biosearch (Petaluma, CA)

| **Species** | **Strain #** | **Source** |
| --- | --- | --- |
| *Escherichia coli* | 10536 | ATCC |
| *Pseudomonas aeruginosa* | 27853 | ATCC |
| *Listeria innocua* | 33090 | ATCC |
| *Campylobacter jejuni* | 33560 | ATCC |
| *Salmonella enterica* | 53648 | ATCC |
| *Legionella pneumophila* SG1 Philadelphia | 33152 | ATCC |
| Lp.1_OLDA | 43109 | ATCC |
| Lp.1_knoxville | 33153 | ATCC |
| Lp.2_Togus -1 | 33154 | ATCC |
| Lp.3_Bloomington-2 | 33155 | ATCC |
| Lp.4_LA-1 | 33156 | ATCC |
| Lp.5_Dallas 1E | 33216 | ATCC |
| Lp.6_Chicago 2 | 33215 | ATCC |
| Lp.7_Chicago 8 | 33823 | ATCC |
| Lp.8_Concord 3 | 35096 | ATCC |
| Lp.9_IN-23-GI-C2 | 35289 | ATCC |
| Lp.10 | 43283 | ATCC |
| Lp.11 | 43130 | ATCC |
| Lp.12 | 43290 | ATCC |
| Lp.13 | 43736 | ATCC |
| Lp.14 | 43703 | ATCC |
| Lp.15_fraseri | 35251 | ATCC |
| *L. birminghamensis* | 43702 | ATCC |
| *L. bozemanii* | 33217 | ATCC |
| *L. dumoffii* | 33279 | ATCC |
| *L. erythra* | Environmental | Environmental |
| *L. feeleii* | Environmental | Environmental |
| *L. gormanii* | 33297 | ATCC |
| *L. hackeliae* | 35250 | ATCC |
| *L. israelensis* | 43119 | ATCC |
| *L. anisa* | 35392 | ATCC |
| *L. jordanis* | 33623 | ATCC |
| *L. longbeachae* | Environmental | Environmental |
| *L. maceachernii* | 35300 | ATCC |
| *L. micdadei* | 33218 | ATCC |
| *L. moravica* | 43877 | ATCC |
| *L. oakridgensis* | Environmental | Environmental |
| *L. parisiensis* | 35299 | ATCC |
| *L. quateirensis* | 49507 | ATCC |
| *L. sainthelensi* | 35248 | ATCC |
| *L. tucsonensis* | 49180 | ATCC |
| *L. wadsworthii* | Environmental | Environmental |
| *A. baumannii* | 19606 | ATCC |
| *B. cepacia* | 25416 | ATCC |
| *C. albicans* | 14053 | ATCC |
| *E. coli* | 25922 | ATCC |
| *E. faecalis* | 29212 | ATCC |
| *K. pneumoniae* | Environmental | Environmental |
| *M. aurum* | Environmental | Environmental |
| *M. fortuitum* | 6041 | ATCC |
| *M. haemophilum* | Environmental | Environmental |
| *M. kansasii* | Environmental | Environmental |
| *M. nonchromogenicum* | Environmental | Environmental |
| *M. porcinum* | 33776 | ATCC |
| *P. mirabilis* | Clinical | Clinical |
| *P.aeruginosa* | 27853 | ATCC |
| *S. epidermidis* | 12228 | ATCC |
| *S. malt* | 51331 | ATCC |
| *S.aureus* | 25923 | ATCC |
| *Serratia marcescens* | 14756 | ATCC |
| *A.hydrophila* | 7966D-5 | ATCC |
| *A.faecalis* | 8750D-5 | ATCC |
| *B.subtilis* | 23857D-5 | ATCC |
| *Clostridium perfringens* | 13124D-5 | ATCC |
| *Flavobacterium johnsoniae* | 17061D-5 | ATCC |
| *K.oxytoca* | 700324D | ATCC |
| *L.monocytogenes* | 19115D-5 | ATCC |
| *P.putida* | 47054D-5 | ATCC |
| *K.aerogenes (aka E.aerogenes)* | 15038D-5 | ATCC |
| *P.protegens (aka P.fluorescens)* | BAA-477D-5 | ATCC |

**Table S2**: Bacterial strains/genomes used in this study.

| **#** | Isolate | Inclusive | | |
| --- | --- | --- | --- | --- |
|  |  | ***L. spp (16s)*** | **Lp 1-15 (*mip)*** | **SG1(*wzm*)** |
| **1** | *L.pneumophila sg.01 phila* | ✓ | ✓ | ✓ |
| **2** | *L.pneumophila sg.01 olda* | ✓ | ✓ | ✓ |
| **3** | *L.pneumophila sg.01 knox* | ✓ | ✓ | ✓ |
| **4** | *L.pneumophila sg.02* | ✓ | ✓ | x |
| **5** | *L.pneumophila sg.03* | ✓ | ✓ | x |
| **6** | *L.pneumophila sg.04* | ✓ | ✓ | x |
| **7** | *L.pneumophila sg.05* | ✓ | ✓ | x |
| **8** | *L.pneumophila sg.06* | ✓ | ✓ | x |
| **9** | *L.pneumophila sg.07* | ✓ | ✓ | x |
| **10** | *L.pneumophila sg.08* | ✓ | ✓ | x |
| **11** | *L.pneumophila sg.09* | ✓ | ✓ | x |
| **12** | *L.pneumophila sg.10* | ✓ | ✓ | x |
| **13** | *L.pneumophila sg.11* | ✓ | ✓ | x |
| **14** | *L.pneumophila sg.12* | ✓ | ✓ | x |
| **15** | *L.pneumophila sg.13* | ✓ | ✓ | x |
| **16** | *L.pneumophila sg.14* | ✓ | ✓ | x |
| **17** | *L.pneumophila sg.15* | ✓ | ✓ | x |
| **18** | *L.anisa* | ✓ | x | x |
| **19** | *L.birminghamensis* | ✓ | x | x |
| **20** | *L.bozemanii* | ✓ | x | x |
| **21** | *L.dumoffii* | ✓ | x | x |
| **22** | *L.erythra* | ✓ | x | x |
| **23** | *L.feeleii* | ✓ | x | x |
| **24** | *L.gormanii* | ✓ | x | x |
| **25** | *L.hackeliae* | ✓ | x | x |
| **26** | *L.israelensis* | ✓ | x | x |
| **27** | *L.jordanis* | ✓ | x | x |
| **28** | *L.longbeachae* | ✓ | x | x |
| **29** | *L.maceachernii* | ✓ | x | x |
| **30** | *L.micdadei* | ✓ | x | x |
| **31** | *L.moravica* | ✓ | x | x |
| **32** | *L.oakridgensis* | ✓ | x | x |
| **33** | *L.parisiensis* | ✓ | x | x |
| **34** | *L.quateirensis* | ✓ | x | x |
| **35** | *L.sainthelensi* | ✓ | x | x |
| **36** | *L.tucsonensis* | ✓ | x | x |
| **37** | *L.wadsworthii* | ✓ | x | x |

**Table S3**: *Legionella* multiplex qPCR assay inclusivity testing. Genomic DNA from 37 listed *Legionella* isolates was tested as PCR template with the GoTaq^®^ *Legionella spp/pneumophila/*SG1 kit. Inclusivity tests were performed at a DNA concentration of 100 GU/well. Exclusivity tests were performed at a DNA concentration of 10,000 GU/well. Green check mark indicates presence of qPCR amplification (C_t_ value<40). Red X indicates lack of qPCR amplification (No C_t_ value called). Raw data file available on request.

| **#** | **Isolate** | ***L. spp***  **(16s)** | **Lp 1-15**  **(*mip1*)** | **SG1**  **(*wzm*)** | **IPC** |
| --- | --- | --- | --- | --- | --- |
| **38** | *A.baumannii* | x | x | x | 26.42 |
| **39** | *B.cepacia* | x | x | x | 26.35 |
| **40** | *C.albicans* | x | x | x | 26.32 |
| **41** | *E.coli* | x | x | x | 26.33 |
| **42** | *E.faecalis* | x | x | x | 26.30 |
| **43** | *K.pneumoniae* | x | x | x | 26.14 |
| **44** | *M.aurum* | x | x | x | 26.18 |
| **45** | *M.fortuitum* | x | x | x | 26.35 |
| **46** | *M.haemophilum* | x | x | x | 26.36 |
| **47** | *M.kansasii* | x | x | x | 26.29 |
| **48** | *M.nonchromogenicum* | x | x | x | 26.37 |
| **49** | *M.porcinum* | x | x | x | 26.25 |
| **50** | *P.mirabilis* | x | x | x | 26.11 |
| **51** | *P.aeruginosa* | x | x | x | 25.92 |
| **52** | *S.epidermidis* | x | x | x | 26.23 |
| **53** | *S.malt* | x | x | x | 26.22 |
| **54** | *S.aureus* | x | x | x | 26.23 |
| **55** | *S.marcescens* | x | x | x | 26.28 |
| **56** | *A.hydrophila* | x | x | x | 26.49 |
| **57** | *A.faecalis* | x | x | x | 26.16 |
| **58** | *B.subtilis* | x | x | x | 26.46 |
| **59** | *C.perfringens* | x | x | x | 26.41 |
| **60** | *F.johnsoniae* | x | x | x | 26.47 |
| **61** | *K.oxytoca* | x | x | x | 26.32 |
| **62** | *L.monocytogenes* | x | x | x | 26.21 |
| **63** | *P.putida* | x | x | x | 26.25 |
| **64** | *K.aerogenes* | x | x | x | 26.37 |
| **65** | *P.protegens* | x | x | x | 26.36 |

**Table S4**: *Legionella* multiplex qPCR assay exclusivity testing. Genomic DNA from 28 listed bacterial isolates was tested as PCR template with the GoTaq^®^ *Legionella spp/pneumophila/*SG1 kit. Exclusivity tests were performed at a DNA concentration of 10,000 GU/well. Green check mark indicates presence of qPCR amplification (C_t_ value<40). Red X indicates lack of qPCR amplification (No C_t_ value called). IPC: Internal Positive Control. Raw data file available on request.

| Sample | *L. pneumophila* vGU/mL | *L. pneumophila vGU/mL* (LOG) |
| --- | --- | --- |
| Unfrozen (+VR) | 18380 | 4.26 |
| Unfrozen (-VR) | 60994 | 4.79 |
| 24h (+VR) | 17573 | 4.24 |
| 24h (-VR) | 69195 | 4.84 |
| 7d (+VR) | 17343 | 4.24 |
| 7d (-VR) | 42200 | 4.63 |
| 1 month (+VR) | 18309 | 4.26 |
| 1 month (-VR) | 35341 | 4.55 |
| EB Unfrozen (+VR) | 0 | - |
| EB 24h (+VR) | 0 | - |
| EB 7d (+VR) | 0 | - |
| EB 1 month (+VR) | 0 | - |

**Table S5**: Frozen stability of VR-treated samples. Paired duplicate samples of cooling tower water samples spiked with *L. pneumophila* were filtered concentrated and treated with/without VR as described in *Materials* *and* *Methods* section. Following neutralization, nucleic acid was extracted immediately (unfrozen) or frozen at -20°C for indicated timeframe, after which nucleic acid was purified, and *L. pneumophila* was quantified using qPCR as described in *Materials* *and Methods.* EB: extraction blank.

**References**

1. Tavernier S, Coenye T. 2015. Quantification of Pseudomonas aeruginosa in multispecies biofilms using PMA-qPCR. PeerJ 3:e787.

2. Bubert A, Köhler S, Goebel WJA, Microbiology E. 1992. The homologous and heterologous regions within the iap gene allow genus-and species-specific identification of Listeria spp. by polymerase chain reaction. 58:2625–2632.

3. Kontchou JA, Nocker A. 2019. Optimization of viability qPCR for selective detection of membrane-intact Legionella pneumophila. J Microbiol Methods 156:68–76.

4. Merault N, Rusniok C, Jarraud S, Gomez-Valero L, Cazalet C, Marin M, Brachet E, Aegerter P, Gaillard JL, Etienne J, Herrmann JL, Group D-IS, Lawrence C, Buchrieser C. 2011. Specific real-time PCR for simultaneous detection and identification of Legionella pneumophila serogroup 1 in water and clinical samples. Appl Environ Microbiol 77:1708–17.

5. Wellinghausen N, Frost C, Marre R. 2001. Detection of legionellae in hospital water samples by quantitative real-time LightCycler PCR. Appl Environ Microbiol 67:3985–93.
